# The Condition Revealed in the Report

**Published:** 1919-10-18

**Authors:** 


### THE DIDWORTHY SANATORIUM INQUIRY.

### The Condition Revealed in the Report.

The Plymouth Insurance Committee, after considering the report of Mr. J. H. Kevern on his inspection of Didworthy Sanatorium, "strongly condemns the arrangements that obtain in that institution, and are (*sic*) of opinion that a sanatorium should be owned and controlled by the municipality." Dr. Wagner received the Chairman's assurance that he, Mr. Kevern, did not feel able to criticise the medical treatment. It was the diet and the comforts to which exception must be taken.

In Mr. Kevern's report criticism fixed on the need for a recreation hut; for a male cook (apparently there has been no cook of either sex for a considerable time); for ware other than enamel at meals, and for the necessary appliances to wash and sterilise it; for more butter and eggs; for the medical examination of patients not earlier than a week before their admission; for the sending of clinical reports and charts to the tuberculosis officer on the patient's discharge; for a hot-carrier from the kitchen; for the "essential motor-ambulance"; for a farm colony, and the payment of patients at work on

**The Didworthy Sanatorium Inquiry**—(*continued*).

the lines adopted at Papworth by the Cambridge After-care Committee; for sterilising the left food before giving it to the pigs, which are eaten by the patients; for reverting to the standard of efficiency in force before 1914; and finally, for treating the patients who are "insured persons" in a municipal sanatorium. The presence of chronic cases, and the consequent delay in receiving others, were also complained of by Dr. Wagner.

Mr. Kevern wisely emphasised the need also for the "control without restriction" of temperamentally difficult patients. We say "wisely" because, though his phrase lacks definition, it implies that a policy is wanted, and it is the lack of such a policy, not to mention the character and experience needed in a medical superintendent to conceive and to enforce it, which has been the chief occasion of sanatorium difficulties.

The Hospital has often insisted that the "heel of Achilles in sanatorium management" is neglect to select patients on temperamental as well as on physical grounds, for it requires brains to be a good patient, particularly if the patient is tuberculous; and some patients are chronically incapacitated temperamentally, as others are chronically unsuitable physically, for admission to a general sanatorium. The chronic, in either case, needs a special institution for his reception. But, so far as the evidence goes, few medical superintendents, the bulk of whom are without long technical experience of sanatorium management, have recognised this. Dr. W. C. Rivers, formerly medical superintendent of Barrasford Sanatorium, and now tuberculosis officer to the West Riding, is a notable exception to the rule. Reforms in diet and so forth, however necessary, are doomed to failure unless the temperamental difficulty is mastered by the governing body and their medical officer. When it has, on the facts, not been mastered, criticisms are made like that of Mr. H. H. Edgington, who declared, at the meeting referred to, that he would rather go to a lethal chamber than to Didworthy as run of late. It remains only to note that a municipal sanatorium may be better than a voluntary one for insured persons, but that it is the management, not the voluntary system, which has been the cause of the present fiasco.

